# The PCR-RFLP-Based Detection and Identification of the *Leishmania* Species Causing Human Cutaneous Leishmaniasis in the Khorasan-Razavi Province, Northeast of Iran

**Published:** 2017-09-08

**Authors:** Anita Mohammadiha, Abdolhossein Dalimi, Mohammad Reza Mahmoodi, Mehdi Parian, Majid Pirestani, Mehdi Mohebali

**Affiliations:** 1 Department of Parasitology and Entomology, Medical Sciences Faculty, Tarbiat Modares University, Tehran, Iran; 2 Department of Microbiology and Parasitology, Faculty of Medicine, Guilan University of Medical Sciences, Rasht, Iran; 3 Department of Medical Parasitology and Mycology, Medical Faculty, Mashhad University of Medical Sciences, Mashhad, Iran; 4 Department of Medical Parasitology and Mycology, School of Public Health, Tehran University of Medical Sciences, Tehran, Iran; 5 Center for Research of Endemic Parasites of Iran (CREPI), Tehran University of Medical Sciences, Tehran, Iran

**Keywords:** Cutaneous leishmaniasis, Human, ITS, Khorasan-Razavi Province, Iran

## Abstract

**Background:**

*Leishmania tropica*, the causative agent of anthroponotic cutaneous leishmaniasis (ACL), and *Leishmania major*, which causes zoonotic cutaneous leishmaniasis (ZCL), are endemic in Iran.

**Methods:**

Cross-sectional study was designed to identify *Leishmania* species in cutaneous leishmaniasis patients who referred to Mashhad Health Centers from 2013 to 2014 using ITS-PCR-RFLP technique. First, physical examinations were performed in all suspected patients and CL cases were confirmed with microscopical examinations. A questionnaire was prepared and completed for each confirmed patient and DNA from each lesion smear was extracted, separately. The ribosomal internal transcribed spacer was amplified with appropriate primers and PCR products were digested by enzyme *Taq1* restrict enzyme.

**Results:**

From all patients, 51 cases (54.3%) were men and 43 of them (45.7%) were women. The most frequent age group was 20–29 years old (27.2%). Hands, face and feet were the most common sites for appearance of skin lesions. All of the 94 cases (100%) tested found to be positive by ITS-PCR-RFLP. Overall, *Leishmania* species were identified in all of the 94 lesion smears which 33 (35%) of them were *L. major* and 61 (65%) of the remained isolates were identified *L. tropica*.

**Conclusion:**

Characterization of *Leishmania* isolates collected from different parts of Khorasan-Razavi Province showed that *L. tropica* is predominant agents of CL, especially in large and medium sized cities such as Mashhad and Shandiz. Moreover, this study revealed that ITS-PCR-RFLP based on our designed primers is a suitable method for species characterization.

## Introduction

Leishmaniasis is caused by parasitic flagellated protozoa of the genus *Leishmania*. CL in Old World is usually caused by *L. major*, *L. tropica*, and *L. aethiopica*. Humans are infected by the bite of infected *phlebotomine* sand flies. Leishmaniasis is a major public health problem with 1.5–2 million new cases annually and with up to 350 million people at risk around the world. Cutaneous leishmaniasis (CL) is currently endemic in 98 countries worldwide (1), and also is still considered an important health problem in some regions of the world, especially the Eastern Mediterranean region, and almost all countries of the Middle East, including Iran (2). In the old world, 90% of cases were reported from Iran, Afghanistan, Saudi Arabia, Iraq, Syria, and Algeria (3). In Iran, the majority of CL is produced by L*. major* while L*. tropica* is only distributed in big and medium cities (4, 5).

The laboratory diagnosis of CL is commonly based on observation of amastigote forms of *Leishmania* in Giemsa stained smears using light microscopy and culture media, but by these methods we cannot identify *Leishmania* species (6, 7). DNA based methods, including various PCR techniques, RFLP analysis and sequencing have been widely used for the identification of *Leishmania* species in humans, animal reservoir hosts and infected vectors (8, 9, 10). Polymerase chain reaction (PCR) assays with high accuracy were used for the detection and species identification of *Leishmania* parasites in humans, animal reservoirs and phelebotomine sand flies.

As Hillis reported in 1991, most rDNA copies seem to be homogeneous, and within each rDNA repeat, there are two internal transcribed spacers (ITS), located between the small subunit (SSU) and large subunit (LSU) rRNA genes (11). ITS1 and ITS2 are separated by the 5.8S rRNA gene, and both provide species-specific sequence markers that have been most frequently detected by Restriction Fragment Length Polymorphism (RFLP) analysis of one-step PCR products (6, 12, 13). Although microscopical technique is applied as gold standard for CL diagnosis, but PCR assay was also used for detection of CL species. We perform a single-PCR-RFLP based on our designed primer (MO) and a nested-PCR-RFLP based on our designed primer (MO) as external primers and ribosomal internal transcribed spacer 1 (ITS1) using primers LITSR and L5.8S (6, 14) as internal primers. Based on prior studies, although ITS1-based primers described previously by El Tai. (14) and Schönian (6), has a perfect positive predictive value (*PPV*), but lacks satisfying sensitivity (15). Sensitivity has been assessed by using Real-time PCR assay (16) or by the serial dilution assay (SDA) (17) based on *Leishmania* serial dilution using cultivated parasites containing known parasite concentrations.

This study attempts to introduce a new ITS-primes for detecting and identifying *Leishmania* species. Furthermore, it maintains amplifying the ITS1 of rDNA (6,14) by designing a Nested-PCR assay as a sensitive method.

## Materials and Methods

### Study design

**Fig. 1.**
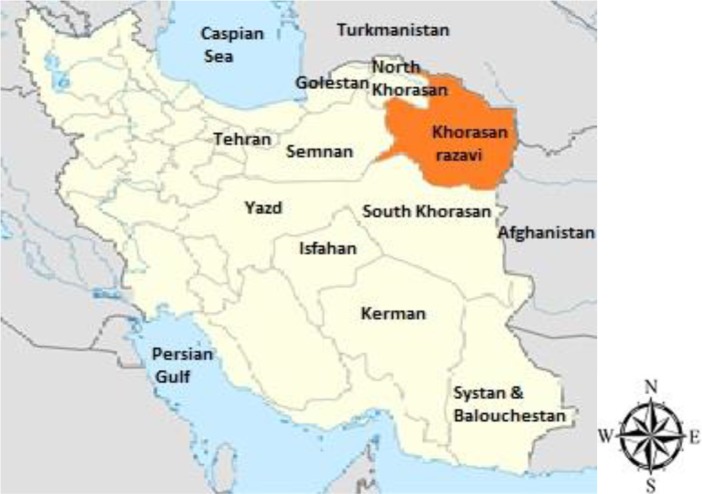
Geographical situation of Korasan-Razavi in the northeast of Iran where the samples were collected

The Khorasan-Razavi Province (36.2980°N 59.6057°E, Fig. 1) located at a mean elevation of 1064m above sea level and covers an area of 144,681km^2^. Its borders are the North Khorasan Province and Turkmenistan in the north, Semnan Province in the west, Yazd and South Khorasan Provinces in the south and Afghanistan and Turkmenistan in the east (Fig. 1).

This study is a descriptive cross-sectional study. The samples were collected from Khorasan-Razavi Province of Iran over a period of 12 months from the summer of 2013 to the summer of 2014 using 94 the suspected cases of CL. All patients were examined by a physician. Different clinical, parasitological and molecular assays were used among CL suspected patients. For each 94 microscopy-confirmed patient of CL, the case’s age and gender, the time between the lesion being first noticed and presentation at a health facility and the location of each lesion on the patient’s body, were recorded. Cutaneous samples (smears) were taken from patients from medical health centers in different leishmaniasis endemic areas of the province, were referred to Parasitology and Entomology laboratory in the Faculty of Medical Sciences of Tarbiat Modares University. At medical health centers, sampling was conducted after gaining accurate information about the place of infection origin. Then after sterilizing around the lesions/nodules with 70% Ethanol, a small incision was made in the margin of the lesion using a disposable lancet and some tissue and exudates were removed by scraping. The scrapings from the lesions were air dried, fixed in methanol, stained with Giemsa 10%, and examined for amastigotes by microscopy.

### Culture of reference strains of *Leishmania*

Reference strains of Old World species of the subgenus *Leishmania* were used: *Leishmania major* (MRHO/IR/75/ER), *Leishmania tropica* (MHOM/IR/99/YAZ1). These strains were taken from *Leishmania* section of School of Public Health and Institute of Public Health Research, Tehran University of Medical Sciences.

They were stored in liquid nitrogen and when necessary, culture was carried out in biphasic culture media (prepared from nutrient agar containing 10% whole rabbit blood overlaid with liver infusion tryptose broth containing 100–200UI/ml penicillin G and 1μg/ml streptomycin). The inoculated cultures were incubated at 21 °C for up to six weeks and examined weekly for the presence of promastigotes. Meanwhile, for mass production of promastigotes, Schneider Insect (HIMEDIA) and RPMI1640 (GIBCO) media were used.

### DNA extraction

First, a slight treatment on the Gimsa-stained slide was done before DNA extraction. Briefly, all slides discolored by incubating in ethanol 100% for 10 minutes, dried at room temperature, then were covered by 1mL distilled water and incubated for 10 minutes at room temperature. The smears removed completely and transferred to a 1.5ml reaction tube, centrifuged at 8,000×g for 5min. Finally supernatant discarded and the pellets were used for DNA extraction.

DNA was extracted with the DNG-plus Extraction Kit (Cinnagen, Iran) according to the manufacturer’s instructions. The DNA pellet was dissolved in 50μL of sterile distilled water and incubated in a water bath at 65 °C for 5min. DNA concentration and quality were determined using Nanodrop ND-1000 Spectrophotometer (Nanodrop Technologies, Wilmington, DE, USA) at 260 and 280nm. DNA samples with A260/A280 ratios between 1.8 and 2 were selected and stored at −20 °C for further analysis.

### PCR-RFLP by MO-ITS-primers

For the first amplification primers were designed based on the ITS region that identified, including: forward primer MO-F: (5′-GCAGCTGGATCATTTTCCGATG-3′) and reverse primer MO-R: (5′-GGCCAACGCGAAGTTGAATTC-3′). The PCR product size stays between 800 and 850bp. The amplification conditions were: 94 °C for 5min, followed by 35 cycles of denaturation at 94 °C for 30s, annealing at 62 °C for 30s and extension at 72 °C for 40s, with a final extension step at 72 °C for 10min.

Restriction fragment length polymorphism (RFLP) analysis of the ITS amplicons was performed on the ITS amplicons, obtained from 94 smear samples and the reference strains, using the restriction enzyme Taq1 (1μL) (Promega, USA) without prior purification. The restriction fragments obtained were compared with the molecular profiles of the WHO reference strains.

**Fig. 2.**
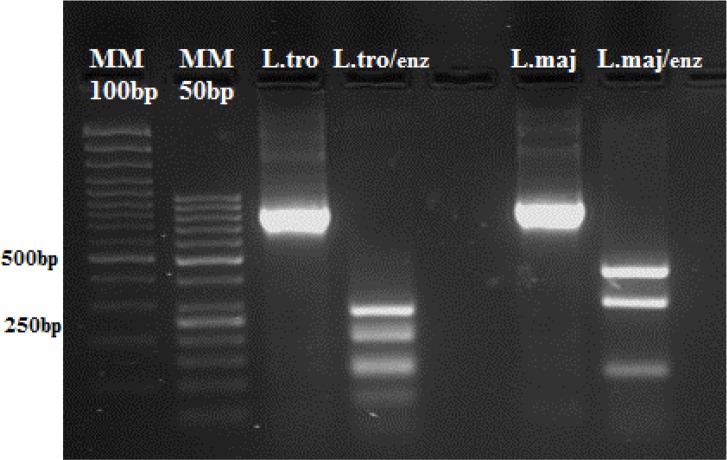
The results of the electrophoresis of the products of the ITS-PCR-RFLP-based amplification of DNA extracted from the stained smears before and after enzymatic digestion. The seven lanes contained a molecular-weight ‘ladder’ 100bp (lane 1) and 50bp (lane 2), the products from reference strains of *Leishmania tropica* before enzymatic digestion (lane 3), after enzymatic digestion (lane 4: fragments of 276, 193, 129, 118, 68 and 28bp, a negative control (lane 5) and *Leishmania major* before enzymatic digestion (lane 6) and after enzymatic digestion (lane 7: fragments of 416, 296, 141 and 26bp)

After using the restriction enzyme, banding patterns were subjected to electrophoresis in 2% agarose (Sigma-Aldrich, St. Louis, MO) at 80V in 1x TAE(40mMTris-acetate, 1mMEDTA, pH8.3) buffer, stained with safe stain (5μL/100mL), and visualized and photographed using a UV transilluminator (Fig. 2).

**Fig. 3.**
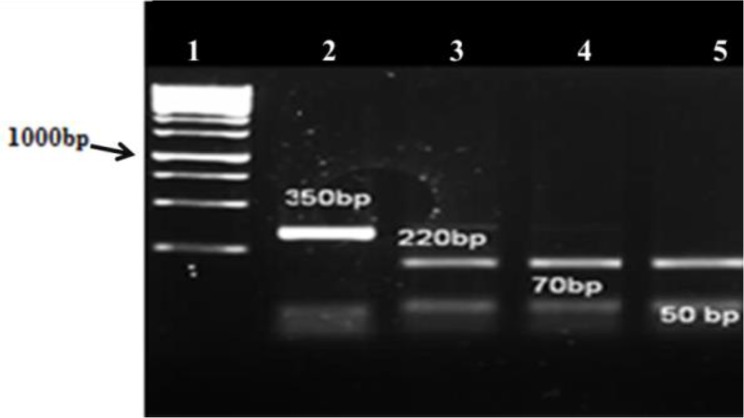
The results of the electrophoresis of the products of the ITS1-PCR-RFLP-based amplification of DNA extracted from the stained smears before and after enzymatic digestion. The five lanes contained a molecular-weight ‘ladder’ 250bp (lane 1), the products from reference strains of *Leishmania tropica* before enzymatic digestion (lane 2), after enzymatic digestion (lane 3, 4 and 5 fragments of 220, 60 and >60bp)

### PCR-RFLP by ITS1-primers

PCR-RFLP was performed as described previously (6) on the 300–360bp fragments amplified from ITS1. The non-purified PCR products (10μl) were digested with 1U of the restriction enzyme *HaeIII* (Promega, Madison, WI, USA), and the restriction fragments obtained were compared with the molecular profiles of the WHO reference strains.

### ITS-n PCR-RFLP

The first amplification performed as mentioned above, based on the ITS region (by MO-ITS primers). For the second amplification 2μL of a 1:20 dilution of the first PCR product was added to 15μL of PCR mix under the conditions as follows: 94 °C for 5min followed by 35 cycles of 94 °C for 30s, 56 °C for 40s, and extension at 72 °C for 1min, followed by a final extension at 72 °C for 5 min. This method was adapted from Schönian et al. (2003) (6). Amplification products were visualized on 2% agarose gel stained with safe stain. The PCR product size stays between 300 and 360bp. The non-purified PCR products (10μl) were digested with 1U of the restriction enzyme *HaeIII* (Promega, Madison, WI, USA), and the restriction fragments obtained were compared with the molecular profiles of the WHO reference strains as are mentioned above.

### *Leishmania* Serial Dilution Assay (SDA) for (Limit of Detection) LOD

Promastigotes from a 4-days-old culture of a reference strain of *L. tropica* (*MHOM/IR/99/YAZ1*), and *L. major* (*MRHO/IR/75/ER*) were washed twice in 1X phosphate-buffered saline and precisely counted on a Neubauer hemocytometer (mean of 10 counts). The DNA was extracted as mentioned above. A series of dilutions was performed, yielding DNA solutions corresponding to decreasing concentrations from 20 to 0.00001 parasite/μl diluted (6 log) by distilled water were used as standard DNA (17).

### Statistical Analysis

The χ^2^-test and Fisher’s exact test using SPSS 16, was used to determine statistically significant differences in disease prevalence between females and males and among different age groups in the community.

## Results

### Characteristics of Patients with Suspected CL.

Fifty one out of 94 (54.3%) of cases were male as well as 43/94 (45.7%) of female cases. Most (>50%) of the suspected cases of CL investigated were aged <28 years. Their age ranges were between 7 months and 78 years. None of the examined patients had been out of their counties during the 6 months preceding the onset of lesions (Table 1).

**Table 1.** Characteristics of features of patients with cutaneous Leishmaniasis in Khorasan-Razavi Province of Iran

**Feature**	**Classification**	**No. of cases**	**Percent**
**Location of lesions**	Face	28	29.8
	Hand	42	44.7
	Foot	19	20.2
	Others	3	3.2
	ND*	2	2.1
	**Total**	94	100

**Gender**	Male	51	54.3
	Female	43	45.7
	**Total**	94	100

**Age**	<10	12	12.8
	10–20	16	17
	20–30	25	26.6
	30–40	12	12.8
	40–50	10	10.6
	>50	17	18.1
	ND*	2	2.1
	**Total**	94	100

ND*: Not Determined

The time between the appearance of the lesion and presentation was 1–4 months for the 73% (16/22) cases infected with *L. major* and 80% (34/42) cases infected with *L. tropica* and not determined for 31 out of 94 (data not shown). The majority of CL lesions, was located on the exposed areas of the body, such as the hand, and then occurs on the face and feet, respectively (Table 1).

### Parasitological results

All of 94 slide samples were found to be positive for the presence of *Leishmania* by microscopy.

### MO-ITS-PCR-RFLP results

After using the restriction enzyme, band patterns, including the fragments of 414, 296, 115 and 26bp for *L. major*, and fragments of 296, 193, 129, 114, 68 and 28bp (actually 4 fragments on gel, 296, 193, 129–114, 68) for *L. tropica* were visualized on electrophoresis gel (Fig. 2). Nucleotide sequence data of *L. major* and *L. tropica* were submitted to the GenBank database with accession no. KP 874100 and KP893242, respectively.

All of 97 cases (100%) tested positive by MO-ITS-PCR-RFLP. *Leishmania major* and *L. tropica* were detected in 33 (35%) of cases and 61 (65%) of cases, respectively by MOITS-PCR-RFLP. The agreement of MO-ITS-PCR-RFLP and Microscopy was 100% (Table 2).

**Table 2.** The numbers of cases of cutaneous leishmaniasis based on *Leishmania* species which were investigated, split by selected cities of Khorasan-Razavi Province

**Species**	**Cities**					

	**Mashhad**	**Sarakhss**	**Shandiz**	**Torghabe**	Others*	**Total (%)**
***L. major***	6	7	1	3	16	33(35%)
***L. tropica***	29	1	2	1	28	61(65%)
**Total**	35	8	3	4	44	94(100%)

* Others: Ahmadabad, Vakilabad, Manzelabad, Ghasemabad, Torbat-e-heidarieh, Dargaz, Torbat-e-jam and Tooss

### ITS1-PCR-RFLP results

After using the restriction enzyme, band patterns, including fragments of 220 and 140 bp for *L. major* and fragments of 200, 60, and <60bp for *L. tropica*, were observed in safe-stained gels.

Fifty five of 94 cases (58%) were detected by ITS1-PCR-RFLP. *L. tropica* was detected by ITS1-PCR-RFLP in 43 of 63 cases (71%), and *L. major* in 12 of 33 cases (36%).

### ITS1-n PCR-RFLP results

Eighty one of 94 cases (86%) tested found to be positive by ITS1-n PCR-RFLP similar to *ITS1-PCR-RFLP* pattern (Fig. 1).

### *Leishmania* serial dilution assay for LOD Results

By conventional PCR, LOD for ITS1-PCR was 1–6 parasites/mL, while the method targeting ITS (MO) could detect 1×10^−2^ parasites/mL.

## Discussion

CL is still considered as an important health problem in many regions of the world, especially in the Eastern Mediterranean region, and almost all countries of the Middle East, including Iran (2, 18). Cutaneous and visceral leishmaniasis both occur in different parts of Iran (19). Where, the prevalence of infection has been reported as 1.8% to 37.9% in different provinces (20, 21). In 2008, 26000 cases in total have been reported and recorded in Iran. More than 90% of cases have happened in 88 cities, and transmission of the disease takes place in the 17 provinces (22).

Khorasan-Razavi Province has common borders with Afghanistan and Turkmenistan in the east, the populations have been increased to 110 percent from 1976 to 2002 and the growth is attributed to the large number of Afghan refugees who constitute a population of approximately 450000 (23). CL is endemic in many parts of Khorasan-Razavi Province (24) and the rate of disease has been increased in various parts of the city in recent years. Mashhad city (the center of Khorasan-Razavi Province) has religious significance (holiest city in Iran), over 20 million pilgrims and passengers visit the city, yearly. Mashhad with 4,900 CL cases (an outbreak of ACL, in 2002) as well as those reported cases is probably underestimated, so that ACL has become the most important endemic disease and has been considered as a health priority (Khorasan Health Centers Reports 2000–2002). Thus, determination of *Leishmania* species seems to be necessary for designing appropriate control programmers (25). Our finding about the species identification are compatible with the results of studies in different regions of Mashhad revealed that *L. tropica* species are dominant (94.2%) in the studied regions of Mashhad City (23, 25, 26, 27).

The most common anatomical location of lesions on patients’ bodies was hands, face, and feet. Since that sand fly can’t bite through shirt, the biting places are mostly, exposed parts of patient bodies to bite during the active season. Furthermore, in most studies, depends on lifestyle and clothing habits this pattern of lesion frequency is seen (28).

Based on our finding about gender there was a significant difference between the number of male cases and the number of female cases (54.3% vs 45.7%), this is probably due to the fact that men are employed outside the home (more than women), or it could be related to their covers. This result is consistent with the results of studies conducted by Mohajery and Dehghani in Mashhad County (29, 30).

The detection methods frequently used for CL (i.e. the microscopic examination of direct smears and/or the culture of biopsies) are not very sensitive and the *Leishmania* species causing each case of CL in Iran is usually only tentatively identified from extrinsic factors, such as the case’s clinical manifestations, time between the appearance of the lesion and presentations and region of residence. In the present study the time between the appearance of the lesion and presentation, unexpectedly, was 1–4 months for all 94 cases infected with both *L. major* and *L. tropica* (data not shown). These results show that traditional methods such as clinical manifestations of the disease are not reliable for the detection of *Leishmania* species, we should turn to alternative methods that have higher sensitivity and accuracy.

The region of Sarakhs, near the border between Iran, Turkmenistan and Afghanistan, the majority of cases were infected by *L.major*. Torghabeh, Shandiz, Sarakhs and Daregaz have the highest disease incidence in the province (31).

Several studies have shown that differences between the agents of CL in the old world (*L. major* and *L. tropica*) may be related to different factors, such as morphological and biological characteristics of the parasite. A variety of molecular, biochemical and immunological methods have been used to characterize and identify the species of *Leishmania*. Currently, the most commonly used method is by PCR. The PCR-RFLP technique revealed that most of examining cases in Mashhad City, Torghabeh, Shandiz, Sarakhss and other cities of Khorasan-Razavi Province were *L. tropica* and *L.major*, providing a suitable focus of ACL and ZCL for further research activities.

A noteworthy result obtained is the determination of *‘Limits of Detection’* (*LOD*) for primer pairs were determined by *‘Serial Dilution Assay’* based-PCR. ITS-based PCR was more sensitive than ITS1-PCR (1–6 parasites/mL) as was previously reported by author (16), while the method targeting ITS (MO) could detect 1×10^2^ parasites/mL. Lachaud et al. (17) designed a similar study with seeded blood sampling with known parasite concentrations and serial dilution assay (SDA). In their study, genomic DNA showed 2–5 parasites/mL, and the highly repetitive kDNA detected 10^−3^ parasites/mL of blood. In 2013, in a similar study, the detection limits for two different targets primer pairs (kDNA-based-PCR and ITS-based PCR) were determined by Taqman-based real-time PCR Assay (16). Therefore the possibility of detection in samples with lower parasitemia using ITS-based PCR was predicted.

In this study, we used ITS1-PCR–RFLP technique for *Leishmania* species identification and our results showed that there was a relatively good concordance was observed between PCR-RFLP technique and parasitological results, where this molecular technique could detect all of the 94 parasitological positive samples (100%).

Despite DNA extracted from all of the 94 samples, the poor results obtained from ITS1-based PCR, it seems that parasite loads within the skin lesions could have an important role in this investigation (16), thus, as suggested in previous studies, the sensitivity of ITS1-based PCR should be improved or it could be used from most sensitive and specific molecular methods, such as nested PCR (32).

## Conclusion

In conclusion, characterization of *Leishmania* isolates collected from different parts of Khorasan-Razavi Province showed that *L. tropica* is predominant agents of CL in Mashhad City and *L. major* is distributed in rural areas and cities which have common borders with neighbor countries. Moreover, this study revealed that ITS-PCR-RFLP based on our designed primers is an appropriate method for characterization of *Leishmania* species.
